# Assessment of Industry-Induced Urban Human Health Risks Related to Benzo[*a*]pyrene based on a Multimedia Fugacity Model: Case Study of Nanjing, China

**DOI:** 10.3390/ijerph120606162

**Published:** 2015-05-29

**Authors:** Linyu Xu, Huimin Song, Yan Wang, Hao Yin

**Affiliations:** 1 State Key Joint Laboratory of Environmental Simulation and Pollution Control, School of Environment, Beijing Normal University, No. 19 Xinjiekouwai Street, Haidian District, Beijing 100875, China; E-Mails: shm8623@mail.bnu.edu.cn (H.S.); wangyan2014@mail.bnu.edu.cn (Y.W.); 201221180031@mail.bnu.edu.cn (H.Y.); 2 China Environment Publishing Co. Ltd., No.16 Guangqumennei Street, Dongcheng District, Beijing 100062, China

**Keywords:** human health risk, environmental exposure, developing countries, rapid industrialization, Nanjing, benzo[*a*]pyrene

## Abstract

Large amounts of organic pollutants emitted from industries have accumulated and caused serious human health risks, especially in urban areas with rapid industrialization. This paper focused on the carcinogen benzo[*a*]pyrene (BaP) from industrial effluent and gaseous emissions, and established a multi-pathway exposure model based on a Level IV multimedia fugacity model to analyze the human health risks in a city that has undergone rapid industrialization. In this study, GIS tools combined with land-use data was introduced to analyze smaller spatial scales so as to enhance the spatial resolution of the results. An uncertainty analysis using a Monte Carlo simulation was also conducted to illustrate the rationale of the probabilistic assessment mode rather than deterministic assessment. Finally, the results of the case study in Nanjing, China indicated the annual average human cancer risk induced by local industrial emissions during 2002–2008 (lowest at 1.99×10^–6^ in 2008 and highest at 3.34×10^–6^ in 2004), which was lower than the USEPA prescriptive level (1×10^–6^–1×10^–4^) but cannot be neglected in the long term.The study results could not only instruct the BaP health risk management but also help future health risk prediction and control.

## 1. Introduction

Rapid industrial development can meet the increasing needs for reliable infrastructure and enhanced living conditions; however, industrialization has also led to numerous health threats. As Higginson [1] discussed, governments and the public need to be aware that the coexistence of newer sophisticated technologies and traditional activities can make the situation more complex. Under these circumstances, an assessment of pollutant exposure and human health risk is essential for more accurate control decisions.

### 1.1. Review of Human Health Risk Assessment Studies

As China has undergone rapid urbanization and industrialization, significant progress has been made in the theories and methodologies applied to the human health risk assessment. Xu and Liu proposed a theoretical information diffusion method for the regional environmental risk assessment to facilitate a more rational approval and construction of industrial sites, as well as a more suitable emergency plan for accidents [2]. Many practical studies were carried out to examine pollutant concentration levels and the resulting health risks in different environmental media, like in air (Xiong *et al*., 2009 [3]; Lin *et al*., 2010 [4]), in water (Zhang *et al*., 2007 [5]); Dong *et al*., 2009 [6]; Li *et al*., 2009 [7]), in soil(Shi *et al*., 2010 [8]; Sun *et al*., 2010 [9]). Most risk assessment practices were based on environmental monitoring data and exposure-risk relationships, hence, the resulting risk estimates were static representations of the past or current situations in the study regions, lacking in long-term trends and future anticipation. For those cities undergoing rapid industrialization, the environmental management measures lag behind rapid industrial development, which leads to increased prominence of industrial pollutant-induced human health risks. Thus, it is necessary to investigate the temporal variations and the spatial distributions of exposure and health risks during rapid industrialization. Multimedia mathematical models of chemicals (e.g., multimedia fugacity models), with proven acceptable estimated accuracy, can be used for further risk estimation as a supplement or alternative to monitoring data (Connell *et al*., 1998 [10]; Ares, 2004 [11]; Yang *et al*., 2005 [12]; Duan *et al*., 2013 [13]). The thermodynamic concept of fugacity was first introduced as an equilibrium criterion among different phases by Lewis in 1901 (Mackay, 1979 [14]). Based on the thermodynamics theory and the mass-balance equations, Mackay *et al*. (2001) discussed the merit and feasibility of the fugacity mode and categorized them into four levels according to system complexity (open or not, equilibrium or not, steady or not), in which Level IV fugacity model is closest to real situation in that it represents an open, unsteady and non-equilibrium system [15]. In addition to providing insights including chemical persistence and long-range transport, it can also generate time-dependent fates of chemicals. Thus, the application of Level IV model can better reflect the nature of chemicals in the environment.

Previous researches have pointed out that polycyclic aromatic hydrocarbons (PAHs) from anthropogenic activities pose non-negligible threats to human health, with more possibilities of causing mutations and cancer. Wu (2010) [16] examined 24 organic pollutants in Nanjing’s drinking water, showing their potential carcinogenic effects. Among all kinds of PAHs, benzo[*a*]pyrene (BaP) is one of the most carcinogenic chemicals (Macleod and Mackay, 1999 [17]), which is categorized as a probable human carcinogen in the 1992 Integrated Risk Information System developed by the United States Environmental Protection Agency (USEPA) and is also listed as a Class One pollutant in the Integrated Wastewater Discharge Standard of China.

Based on the above review, in this study, we focused on the health risks induced by BaP release due to rapid industrialization and urbanization in Nanjing, China. By utilizing a Level IV fugacity model to simulate the fate of chemicals and multi-pathway exposure model to assess public exposure level, we generate the variation of human health risks with time and space. We also carried out an uncertainty analysis using Monte-Carlo simulation to get the probability distribution of the risks. All the work is conducted with the intention of assisting future urban health risk management, especially in fast urbanization areas.

### 1.2. Study Area

**Figure 1:**
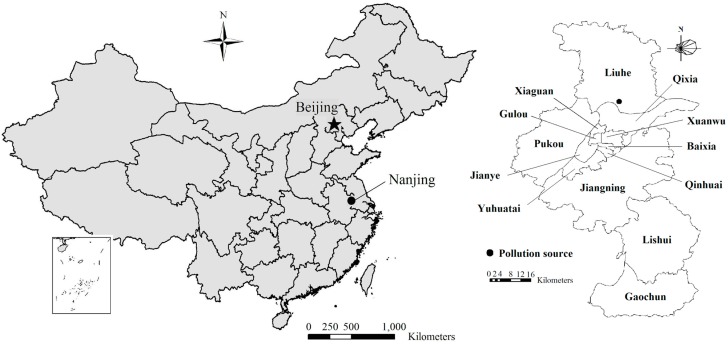
The location and administrative districts of Nanjing in China.

Nanjing is a historic city and capital of Jiangsu Province in China. It is located along the Yangtze River (Figure 1) and has an average annual temperature of 15.4×Cand 1000 mm precipitation. The city has undergone rapid industrialization and urbanization in recent years. The gross industrial output increased with an annual average pace of over 17%from 2000 to 2010 (Nanjing Statistics Bureau, 2011) [18].

New industrial development plans at both the local and regional levels have been carried out. Most notably, the Nanjing Chemical Industry Park (NCIP) was built in 2001 as the only petrochemical industry base approved by the nation in Jiangsu Province (Nanjing Planning Bureau, 2001) [19].With an area of 45 km^2^, it hosts well-known national and international plants dedicated to industries such as petroleum refining and the manufacture of natural gas chemicals, organic raw materials and fine chemical products.

## 2. Materials and Methods

### 2.1. Spatial Data Extraction

In this study, in order to improve the assessment accuracy, we combined geographic information system (GIS) data with the human health risk assessment and health risk management. The whole process works in the following manner:

(1) Determine the boundaries of the study area (in this case the administrative boundaries of Nanjing), collect the industrial pollution emissions inventory in GIS format (including location, concentration and volume), and identify chemicals and emission sources.

(2) Interpret remote sensing images, extract relevant environmental parameters (such as the underlying surface conditions) and spatial information for the fugacity model.

(3) Due to different underlying substrates, the study area is divided into a series of sub-regions that are connected to each other.

(4) Then we established the mass balance differential equations, and the material exchange relationships among various sub-regions.

(5) Set the initial value of the parameters (such as fugacity), run a dynamic multi-media fugacity model to predict pollutant concentrations in environmental media in the different sub-regions.

(6) Calculate risk values based on the estimated concentration, multi-channel exposure model and dose-risk relationships.

(7) The risk assessment results are demonstrated in GIS format, to reflect whether health risk in the sub-regions is higher than the prescriptive value, and discuss how to reduce the risk.

### 2.2. Pollutant Concentration Calculation

Level IV fugacity model uses differential equations to write mass balance equations within each medium so these equations can be integrated to give the fugacity as a function of time, thus quantifying the time response characteristics of the system (Mackay, 2001) [15].

**Figure 2:**
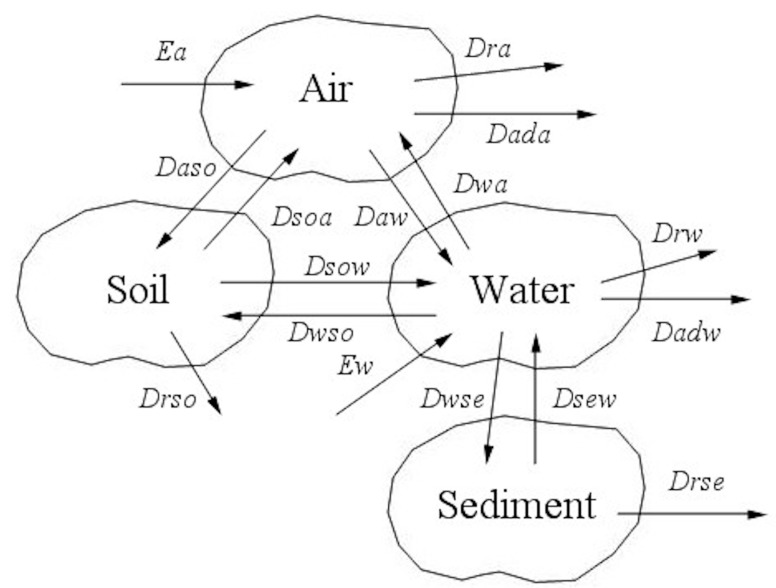
Chemical transport processes in the environment.

As Figure 2 shows, air, water, soil and sediment compartments and their respective sub-compartments were considered. The transportation processes include interface transport (diffusion, deposition, dissolution, runoff and resuspension) and non-interface transport (advection, deterioration) as well as anthropogenic emission. Chemical fugacity is in equilibrium within different phases of each compartment but is in non-equilibrium among the four compartments. Model calculation is accomplished using the AnyLogic software (The AnyLogic Company, Saint-Petersburg, Russia, http://www.xjtek.com/).

Pollutant emissions (BaP) from both industry and transportation were considered in the validation of the multimedia fugacity model and risk assessment. Considering that industry pollutants account for the most part of pollution in Nanjing (industry, 63.9%; transportation, 31%) (Ding, 2004) [20] and it is also the key urbanization characteristic we consider in this paper, this was further considered in the uncertainty analysis. The industrial BaP emissions to the air and water compartments were estimated from the emission quantity (*Nanjing Statistical Yearbook)* and chemical concentration (assumed to satisfy relevant national discharge standards). The emission rate from transportation (Table 1) was estimated based on the source apportionment results (Ding, 2004) [20]. Other pollution sources, such as chemical advections from outside areas into the study area, were considered to be constant to reflect the human health risk induced by new local industrial emissions during a specific period.

**Table 1:** Estimated emission rates of BaP (2002–2008, mol/day).

Media	Source	2002	2003	2004	2005	2006	2007	2008
Air	Transportation ^a^	0.43	0.50	0.51	0.59	0.62	0.64	0.69
	Industry ^b^	0.89	1.04	1.06	1.22	1.28	1.32	1.43
	Total	1.32	1.54	1.58	1.82	1.90	1.96	2.12
Water	Industry ^b^	0.17	0.16	0.15	0.15	0.14	0.13	0.12

Notes: ^a^ (Ding, 2004); ^b^ *Nanjing Statistical Yearbook* (In Chinese), *Integrated Emission Standard of Air Pollutants* (GB 16297–1996, in Chinese) and *Integrated Wastewater Discharge Standard* (GB 8978–1996, in Chinese).

Several characteristics of rapidly industrialized areas were considered in this study. First, agricultural irrigation is the major route of chemical transport from the water compartment to the soil compartment in regions where agricultural production is still an important contributor to the local economy. Thus, the chemical transport from water to soil (*D_wso_*) was added (Equations (2,3)). Second, built-up areas (a term referred to as “urbanized regions” in statistical data of China) were considered because artificial impermeable surfaces separate the air and soil compartments and reduce the contact area between them. This factor should not be ignored, especially in regions where built-up areas expand rapidly. The Level IV multimedia fugacity model used here comprises the following differential equations:

(1) $$\text{Air}\;\frac{df_{a}}{dt}=\frac{E_{a}+G_{a}c_{a}+f_{w}D_{wa}+f_{so}D_{soa}-f_{a}\left(D_{aw}+D_{aso}+D_{ra}+D_{ada}\right)}{V_{a}Z_{a}}$$

(2) $$\text{Water}\;\frac{df_{w}}{dt}=\frac{E_{w}+G_{w}c_{w}+f_{a}D_{aw}+f_{so}D_{sow}+f_{se}D_{sew}-f_{w}\left(D_{wa}+D_{wso}+D_{wse}+D_{rw}+D_{adw}\right)}{V_{w}Z_{w}}$$

(3) $$\text{Soil}\;\frac{df_{so}}{dt}=\frac{f_{a}D_{aso}+f_{w}D_{wso}-f_{so}\left(D_{soa}+D_{sow}+D_{rso}\right)}{V_{SO}Z_{SO}}$$

(4) $$\text{Sediment}\;\frac{df_{se}}{dt}=\frac{f_{w}D_{wse}-f_{se}\left(D_{sew}+D_{rse}\right)}{V_{se}Z_{se}}$$

where *a*, *w*, *so* and *se* denote the air, water, soil and sediment compartments, respectively; *f_i_* is the chemical fugacity in compartment *i* (Pa); *t* is the time (day); *E_i_* is the chemical emission to compartment *i* (mol/d); *G_i_* is the inflow rate of compartment *I*(m^3^/day); *c_i_* is the chemical concentration in the inflow of compartment *i*(mol/m^3)^; *D_ij_* is the chemical transport from compartment *i* to compartment *j*(mol/(Pa·day)); *D_ri_* is the chemical degradation in compartment *I*(mol/(Pa·day)); and *D_adi_* is the advection of compartment *i* to the outside areas (mol/(Pa·day)). *V_i_* is the volume of compartment *i* (m^3^); and *Z_i_* is the fugacity capacity of compartment *I* (mol/(m^3^·Pa)).

Details of the variables above and the annual average values of environmental variables are shown in the Supplementary Information. The daily data of meteorological parameters were obtained from the *China Meteorological Data Sharing Service System* (http://cdc.cma.gov.cn/). The daily varied air advection was estimated from wind speed, wind direction and other local parameters. Due to a lack of local environmental data, typical values from Mackay (2001) [15], including the volume fractions of different phases in the four compartments, the molecular diffusion coefficients among different compartments and so on, were used for the other variables not presented in the Supplementary Information. The initial values of the BaP concentration in the environmental compartments and inflows were considered in the same order of magnitude with those reported in the literature. The initial BaP fugacity in the air, water, soil and sediment compartments were assumed at an order of magnitude of 1×10^–11^, 1×10^–10^, 1×10^–11^ and 1×10^–10^, respectively. The BaP concentrations in the air and water inflows were set to 1×10^–11^ and 1×10^–7^mol/m^3^, respectively.

### 2.3. Health Risk Exposure Assessment

Ingestion of food and tap water, inhalation and dermal contact were considered as the four major exposure pathways; therefore, in this study, we utilized a multi-pathway exposure model to do the exposure assessment. An average exposure level was considered in this study. It was assumed that all of the local agricultural products were consumed by local residents and that imported food was free of pollutants.

The chemical exposure dose from food (*D_f_*) was calculated in fugacity form using Equation (5). The pollutant concentration in each food item was estimated by multiplying the fugacity with the fugacity capacity of that food item. McLachlan (1996) [21] concluded that the fugacity of moderately hydrophobic and moderately involatile persistent chemicals remains relatively constant throughout the agricultural food chain. Both biomagnifications and “biodilution” effects along the food chain have been observed in field investigations. Specifically, PAH concentrations decrease along the marine food chain, which may be a result of rapid PAH metabolism or assimilation in organisms at higher trophic levels (Nifon *et al.*, 2008 [22]; Takeuchi *et al.*, 2009 [23]). Considering previous research findings and uncertainty regarding the food web in this study, fugacity in fish was assumed to equal to that of the water compartment, and the fugacity in other food items equals the predicted air fugacity:

(5) $$D_{f}=\frac{1000\times M\times K_{ow}\times Z_{water}\times \left(f_{a}\times \sum \left(v_{k}\times m_{k}\right)+f_{w}\times v_{fish}\times m_{fish}\right)}{\rho_{lp}\times W}$$

Where *D_f_* is the exposure dose from food (mg/(kg·day)); *M* is the chemical molar mass (g/mol); *K_ow_* is the octanol-water coefficient of the chemical; *Z_water_* is the fugacity capacity of the water phase (mol/(m^3^·Pa)); *v_k_* and *v_fish_* are the mass fraction of lipids in food item k and fish, respectively; *m_k_* and *m_fish_* are daily consumption of food and fish (g/day), respectively; *ρ_lp_* is the density of lipid (g/m^3^); and *W* is the average body weight (kg).

The exposure dose from tap water (*D_w_*), inhalation (*D_i_*) and dermal contact with air and shower water (*D_d_*) were estimated using Equatopms (6)–(8). Suspended solids in the surface water were considered to be removed during the drinking water treatment:

(6) $$D_{w}=1000\times f_{w}\times Z_{water}\times M$$

(7) $$D_{i}=10^{9}\times f_{a}\times Z_{a}\times M$$

(8) $$D_{d}=\frac{1000\times f_{a}\times Z_{a}\times M\times S_{a}\times K_{p}}{W}+\frac{1000\times f_{w}\times Z_{water}\times M\times S_{a}\times K_{p}\times T_{s}}{W}$$

where *D_w_* is the exposure dose from tap water (μg/L); *D_i_* is the exposure dose from air inhalation (ng/m^3^); *D_d_* is the exposure dose from dermal contact (mg/(kg·day)); *S_a_* is the average dermal surface area (m^2^); *K_p_* is the dermal permeability coefficient (m/day); and *T_s_* is the average shower time per day (day/day).Model variables for exposure dose estimates are listed in the Supplementary Information.

### 2.4. Health Risk Characterization

Risk characterization is a process in which risk levels are quantified through relationships between exposure dose and risk. Linear extrapolations of low-dose risk relationships from those at higher exposure concentrations have been extensively used in the current guidelines for cancer risk assessments without adequately evaluating whether a linear low-dose extrapolation is appropriate (Borak and Sirianni, 2005) [24]. U- or J-shaped dose-response relationships, which reflect the existence of the hormesis effect, have been reported in numerous well-designed studies of various biomarkers of carcinogenesis (Calabrese and Baldwin, 1998) [25]. However, the hormesis effect has not been extensively proved for most carcinogenic chemicals. Specifically, the hormesis effect of BaP has not been confirmed, and only one report (Hose and Puffer, 1984) [26] mentioned the possible existence of hormesis. Hence, linear patterns using slope factors or unit risk values published in previous reports were applied in this study. The total human health risk is the sum of the risks from food ingestion, tap water, inhalation and dermal contact. A human health risk of 1.0×10^–6^ means that out of one million people that are exposed to industrial pollutant emissions in the study area, one of those may develop cancer because of such an exposure:

(9) $$R_{f}=D_{f}\times SF_{f}$$

(10) $$R_{w}=D_{w}\times UR_{w}$$

(11) $$R_{i}=D_{i}\times UR_{i}$$

(12) $$R_{d}=D_{d}\times SF_{d}$$

(13) $$R_{t}=R_{f}+R_{w}+R_{i}+R_{d}$$

Where *R_f_*, *R_w_*, *R_i_* and *R_d_* are the risks posed from food ingestion, tap water, inhalation and dermal contact, respectively; *R_t_* is the total cancer risk; *SF_f_* and *SF_d_* are the slope factors of food ingestion and dermal contact, respectively (per mg/(kg·day)); *UR_w_* is the unit risk of tap water (per·μg/L); and *UR_i_* is the unit risk of inhalation (per·ng/m^3^).

### 2.5. Uncertainty Analysis

In the 1940s, Monte Carlo simulation was developed as a computer-based analysis method to estimate uncertainties and variabilities (USEPA, 1997) [27].The EPA has pointed out that the probability density functions from Monte Carlo analysis and other analysis techniques can provide adequate supporting data and credible assumptions that could be used as variability and uncertainty analysis of practical statistical tools in risk assessment. This study evaluated parameter uncertainty in risk assessment by Monte Carlo simulation.

Firstly, we utilized the relative sensitivity to identify the key input parameters. The relative sensitivity ($\bar{S}$) is defined as the ratio of the relative change of the modeled result to that of the input parameter, as Equation (14) shows. Each parameter is multiplied by factors (0.9 and 1.1) to get the variation of the result, choosing the parameters that influence the model results most significantly.

(14) $$\bar{S}=\frac{\Delta R/R}{\Delta \overset{t}{\alpha /}\overset{t}{\alpha }}$$

where $\bar{S}$ is the relative sensitivity caused by parameter  α, Δα and $\text{Δ}R_{t}$ refer to the variation of parameter α and modeling results $R_{t}$.

The probability density functions of the most sensitive parameters were estimated based on literatures and local statistical data. These probability density functions of model input were used in the Monte Carlo simulation to analyze the model uncertainty. Uncertainty induced by some of these parameters can also be obtained by conducting Monte Carlo simulation of certain group of parameters, such as environmental parameters and exposure parameters. The results of Monte Carlo simulation and the point estimate can be combined to provide more information about the uncertainty of the model output.

## 3. Results and Discussion

### 3.1. Validation of the Multimedia Fugacity Model

The Level IV multimedia fugacity model was run in AnyLogic considering important emission sources (industry and transportation), background and inflow concentrations. The results in Table 2 indicated that the simulated concentrations of the four compartments were in the range of measured values, like simulated BaP concentration in 2007 is 0.025μg/L in water and16.00 ng/g in soil, both of which lay in the measured level of 0.015–0.033μg/L (or 0.029–0.089μg/L) and <34.31 ng/g with an average of 2.21 ng/g. What’s more, the concentration levels in soil and sediment are 10–100 fold those in air and water, which is in accordance with the fact that soils and sediments contain higher organic contents. All these confirmed that the model was reliable for an environmental concentration prediction.

**Table 2:** Comparison of the measured and the simulated concentrations of BaP in Nanjing.

Compartment	Measured				Simulated		Unit	Literature
	Min	Mean	Max	Year	Mean	Year		
Air	0.962	5.1	31.6	2002–2003	2.34	2002	ng/m^3^	[20]
	0.41	4.17	19.4	2001–2002				[28]
Water	0.015	0.024	0.033	2007	0.025	2007	μg/L	[29]
	0.029	0.043	0.089	2007				[30]
Soil	ND ^a^	2.21	34.31	Not specified	16.00	2007	ng/g(dry)	[31]
Sediment	1.94	22.95	34.85	Not specified	15.04	2002	ng/g(dry)	[32]

^a^ ND = Under detection limit.

### 3.2. Temporal Variations of Urban Human Health Risk Induced by Industrial Pollutant

The fugacity in all four compartments ranged from 10^–14^–10^–13^ Pa. The fugacity in water exhibited a stepwise variation, which may be a result of yearly specific variables. Fluctuations of fugacity in water within a year may be related to a higher ambient temperature and a more intensive rain rate in the summer. Fugacity in the air fluctuated at a relatively low level, but the values for the soil and sediment compartments steadily increased.

The annual average concentrations in the soil and sediment compartments steadily increased to levels several times greater than those at the beginning, but those in the air and water compartments fluctuated within a small range. The concentrations in the soil and sediment compartments were clearly higher than those in the air and water compartments, which may be a result of the lipophilicity of BaP and the higher organic matter content in the soil and sediment.

**Figure 3:**
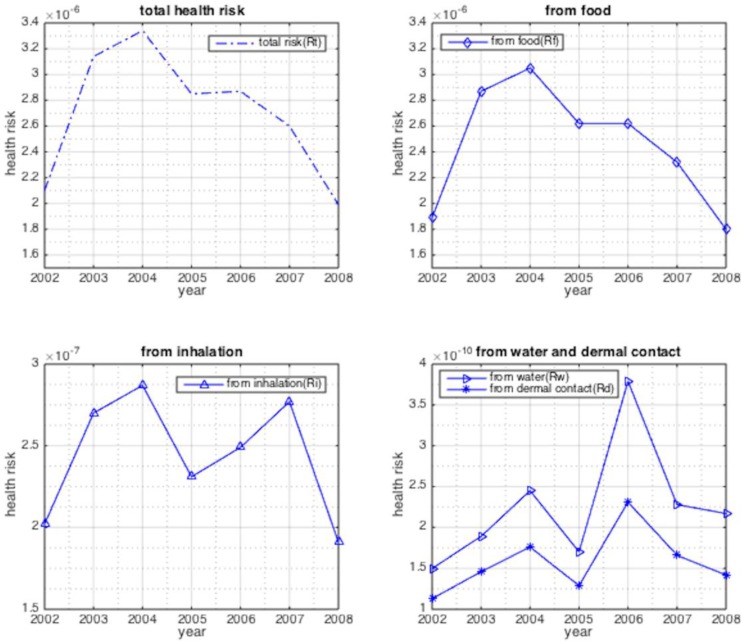
The annual average cancer risk from different pathways from 2002 to 2008.

Exposure doses and the corresponding cancer risks from four pathways were estimated according to Equations (5)–(13). The results from Figure 3 illustrated that magnitude of the annual average cancer risk from the above four pathways were 10^–6^, 10^–10^, 10^–7^, 10^–10^ respectively, indicating the main carcinogenic route is from food ingestion.

The magnitude of annual average cancer risk (*R_t_*) from the sum of the four pathways was 10^‒6^, meaning about one people out of 10^6^ may develop cancer due to BaP exposures from industries and transport emissions in Nanjing each year. In addition, *R_t_*increased from 2002 to 2004 and then decreased to 1.99×10^–6^, taking the form of fluctuation within a range. This may be a result of parameter variations, such as seasonal and daily changes in ambient temperature.

The range of 1×10^–6^–1×10^–4^ was used as a theoretical value for an acceptable lifetime cancer risk by the USEPA (2000) [33] and was used in this study because no relevant regulation has been published in China. Human cancer risks posed by emitted BaP from 2002 to 2008 in Nanjing fluctuated between the lowest and highest bounds, which meant that these risks could cause adverse carcinogenic effects for Nanjing residents at a level that may be acceptable now, but considering the fluctuation and future accumulation, the health risks cannot be neglected in the long term.

### 3.3. The Spatial Distribution of Urban Human Health Risk Induced by Industrial Pollutant

We previously discussed how to combine the multimedia fugacity model and a spatial analysis tool (e.g., Geographic Information System, used for analyzing land use data and spatially presenting the risk assessment results) by proposing procedures and presenting an example in a hypothetical region (Song and Xu, 2011) [34]. The major procedures include collecting data, dividing the study area into several sub-regions, establishing differential mass balance equations of each sub-region, setting parameter values, running the model and analyzing the results. The same method was applied to Nanjing to study the spatial distribution of urban human health risk induced by an industrial pollutant such as BaP.

According to the administrative borders, the city was divided into 13 districts (Figure 1). It was assumed that the emission source was located in the NCIP in the Liuhe District in north Nanjing near the Yangtze River. BaP transport through the air or water compartments among different districts was added in the mass balance equations for the four compartments of the 13 districts. Due to the fact that the land-use types influence the pollutants transportation process, in this study, we collected land use data obtained by interpreting remote sensing images taken in April 2006. Wind direction influences the pollutants transportation direction, especially in the atmosphere. In this study, the prevailing wind direction in Nanjing is from the east or southeast in the summer and from the east or northeast in the winter. Here, an east wind direction was selected. The local values of the other parameters in 2006 were adopted to be consistent with the land use data.

**Figure 4:**
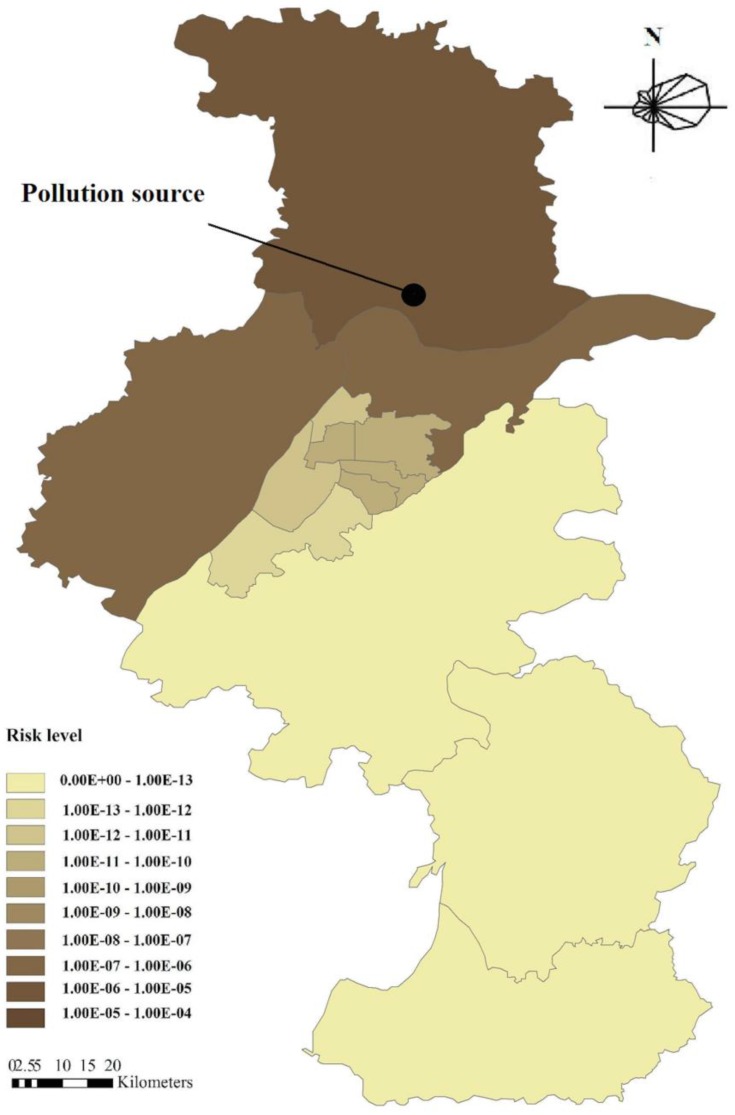
The spatial distribution of the cancer risk induced by industrial BaP in Nanjing in 2006 with east wind.

When the wind blew from the east, BaP emitted in the Liuhe District had little influence on the urban areas of Nanjing (Figure 4). This is mainly a result of the reasonable location of the pollution source (NCIP). In this situation, BaP cannot be directly transported by the wind to urban areas. It may, however, be transported among different compartments of nearby districts and into the Yangtze River and may consequently transport in the water flow and possibly influence the environment in urban areas. The situation may be more serious under other wind directions (for example, the pollutants may transport through the air into the urban areas when the wind blows from the north) and should be investigated.

### 3.4. Uncertainty Analysis

#### 3.4.1. Qualitative Analysis of the Model Uncertainty

The uncertainties in human health risk analysis in this study arise from numerous aspects. First, the industrial BaP emissions in Nanjing were calculated according to the discharge standard and statistical data. Second, data for environmental parameters were not recorded in the early days, so some average values or typical values from the literature were used as surrogates for the fugacity estimates. Third, the average human exposure dose was estimated. These uncertainties arise from random sampling errors (Frey and Zhao, 2004) [35]. To quantify these uncertainties, representative sample data sets for each variable are needed in an exposure assessment. Fourth, the evidence of the hormesis effect of BaP is still insufficient, so linear exposure-response relationships based on slope factors or unit risks were used in risk estimation. Therefore, future research could investigate the hormesis effect in chemical cancer risk assessments, especially for developing countries that require efficient investment approaches during rapid industrialization.

#### 3.4.2. Sensitivity Analysis

Relative sensitivity functions were used to calculate the sensitivity of the model output (*R_t_*) to variations in each parameter of the risk assessment process. The model parameters with the greatest influence on the output were categorized into three groups (Supplementary Information).Group 1 is environmental and physicochemical parameters(some of which may be correlated) used to estimate the concentration, Group 2 is used to estimate the exposure, and Group 3 is used for the risk assessment (Table 3).

**Table 3:** Results of relative sensitivity analysis.

Factor	E_a_	G_a_	T	v_aero_	S	K_ow_	W	SF_f_
1.1α ^a^	92.82%	−79.93% ^b^	84.66%	−77.35%	87.24%	87.24%	−83.80%	91.96%
0.9α	93.25%	−96.26%	85.09%	−94.11%	85.09%	85.52%	−102.28%	92.39%
Group ^c^	1	1	1	1	1	1	2	3

^a^ α is any parameter of the model (e.g., *S* and *K_ow_*); ^b^ Negative values indicate a change in the opposite direction; ^c^ Group 1 is environmental and physicochemical parameter(some of which may be correlated), Group 2 is used for exposure estimation, and Group 3 is used for risk assessment.

#### 3.4.3. Monte Carlo Simulation for the Parameter Uncertainty Analysis

Probability density functions (PDFs) of the previously discussed eight parameters were estimated from the literature. Triangular distribution has been usually used in risk assessment when few data were available to fit the parameter distribution (Wang *et al*., 2009) [36]. As a result of the independence among the parameters used for the concentration estimation (Group 1), the PDFs of these parameters were not estimated separately. Instead, point estimates of BaP concentrations were calculated and used to estimate the PDFs in the uncertainty analysis. It was assumed that the concentrations follow a triangular distribution as (0.5α, α, 1.5α), where α is the mode. PDFs of *W* and *SF_f_* were estimated from a USEPA report (1997) [27] where point estimates were presented. Food ingestion data in 2005 were taken as an example for the uncertainty analysis. The distributions used for 2005 are listed in the Supplementary Information.

**Figure 5:**
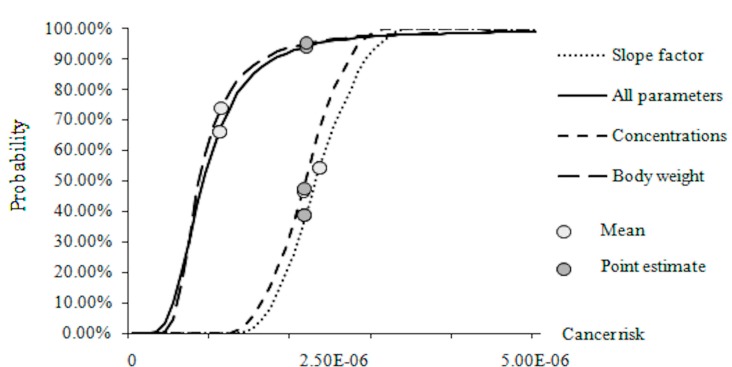
The probability cumulative distribution functions (CDFs) of the cancer risk in 2005.

A Monte Carlo simulation was performed for Groups 1, 2, and 3 and in a combination of all three groups (each 5000 times). Cumulative distribution functions (CDFs) for the risk in 2005 under these conditions are presented in Figure 5.

When the uncertainties induced from all parameters are considered, the distribution skewed from the point estimate value (Figure 5). The point estimate of the risk from food ingestion (2.14×10^–6^) in 2005, which was within the acceptable range, was higher than most of the values in the distribution function. This meant that the results for the probabilistic assessment may indicate risks below the lower bound of the acceptable range. The situation was different, however, when it considered for different groups in the form of a PDF separately. The point estimate of the body weight may overestimate the risk, whereas that of the slope factor may underestimate the risk. Point estimate and average values for the cancer risk caused by BaP concentrations were close. In summary, the introduction of the probabilistic assessment mode may provide more information about the risk and contribute to risk management more significantly than the deterministic assessment.

## 4. Conclusions

The multimedia fugacity model is an appropriate compensation to most health risk assessments, which mainly rely on available monitoring data in that it can simulate the fate of chemicals on the basis of environmental and chemical characteristics., so this multimedia mathematical model can be used to predict the chemical fugacity in scenarios that may occur in the future to keep up with the fast pace of urbanization. In this study, the fugacity in water exhibited a stepwise variation, the fugacity in the air fluctuated at a relatively low level, and the fugacity in the soil and the sediment compartments steadily increased, depicting a general picture of chemical concentration in different environmental media.

The magnitude of the annual average human health risks from food ingestion, tap water, inhalation and dermal contact were 10^–6^, 10^–10^, 10^–7^ and 10^–10^, respectively, so food ingestion is the most important exposure pathway for BaP in Nanjing, China. The estimated values also suggested that a slightly decreasing trend for the cancer risk from food ingestion and inhalation, but the risks from tap water and dermal contact increased. Hence, it is appropriate to consider reduction measures for risks from food ingestion in future risk management of BaP.

The annual average human health risk posed by industrially discharged BaP from the four pathways fluctuated during 2002–2008. The risk values were within the low and high boundaries of the prescriptive range, which means that BaP related health risk could cause adverse carcinogenic effects to Nanjing residents at a level that may be acceptable now. But due to accumulation and annual fluctuations, the risks cannot be neglected in the long term.

The results of the spatial analysis of the urban human health risk induced by an industrial pollutant may be further used for industrial pollution emission control, including evaluating both current emission sources and those that will emerge in the future.

The uncertainty analysis using a Monte Carlo simulation differentiate among the roles of different factors in influencing health risks, indicating that the introduction of the probabilistic assessment mode may provide more information about risks and contribute to risk management more significantly than the deterministic assessment.

## Supplementary Files

Supplementary File 1
