# Supplementary material for: Assessment of Industry-Induced Urban Human Health Risks Related to Benzo[a]pyrene based on a Multimedia Fugacity Model: Case Study of Nanjing, China
Source: Int J Environ Res Public Health. 2015 May 29;12(6):6162–78. doi: 10.3390/ijerph120606162 (PMC4483694; doi:10.3390/ijerph120606162)
Supplement: Supplementary File 1 [file ijerph-12-06162-s001.pdf]

Supplementary Information

Assessment of Industry-Induced Urban Human Health Risks Related to Benzo[*a*]pyrenebased on a Multimedia Fugacity Model: Case Study of Nanjing, China

---

Details of the Variables in the Four Mass Balance Equations

Variables in Equations (1)–(4) were calculated in the following equations which were mainly from previous studies by other researchers, especially from the publication by Mackay in 2001. Built-up areas and chemical transport from the water compartment to the soil compartment were also considered in this study.

D values were estimated from the following equations:

$$D_{aw} = \frac{1}{\frac{1}{K_{awa} \times A_{aw} \times Z_{air}} + \frac{1}{K_{aww} \times A_{aw} \times Z_{water}}} + (A_{aw} + A_{ur}) \times U_r \times Z_{water} + (A_{aw} + A_{ur}) \times U_r \times Q \times v_{aero} \times Z_{aero} + A_{aw} \times U_{dd} \times v_{aero} \times Z_{aero} \quad (1)$$

$$D_{wa} = \frac{1}{\frac{1}{K_{awa} \times A_{aw} \times Z_{air}} + \frac{1}{K_{aww} \times A_{aw} \times Z_{water}}} \quad (2)$$

$$D_{aso} = \frac{1}{\frac{1}{K_{asoa} \times A_{aso} \times Z_{air}} + \frac{L_{so}}{A_{aso} \times B_{ma} \times Z_{air} + A_{aso} \times B_{mw} \times Z_{water}}} + A_{aso} \times U_r \times Z_{water} + A_{aso} \times U_r \times Q \times v_{aero} \times Z_{aero} + A_{aso} \times U_{dd} \times v_{aero} \times Z_{aero} \quad (3)$$

$$D_{soa} = \frac{1}{\frac{1}{K_{asoa} \times A_{a\sigma o} \times Z_{air}} + \frac{L_{so}}{A_{aso} \times B_{ma} \times Z_{air} + A_{aso} \times B_{mw} \times Z_{water}}} \quad (4)$$

$$D_{wso} = G_{wso} \times Z_w \quad (5)$$

$$D_{sow} = A_{aso} \times U_{soff} \times Z_{soil} + A_{aso} \times U_{woff} \times Z_{water} \quad (6)$$

$$D_{wse} = \frac{1}{\frac{1}{K_{wsew} \times A_{wse} \times Z_{water}} + \frac{L_{se}}{A_{wse} \times B_{mse} \times Z_{water}}} + A_{wse} \times U_{de} \times Z_{sus} \quad (7)$$

$$D_{sew} = \frac{1}{\frac{1}{K_{wsew} \times A_{wse} \times Z_{water}} + \frac{L_{se}}{A_{wse} \times B_{mse} \times Z_{water}}} + A_{wse} \times U_{re} \times Z_{sedi} \tag{8}$$

$$D_{ri} = \frac{0.693 \times V_i \times Z_i}{\tau_{1/2i}} \tag{9}$$

$$D_{adi} = G_i \times Z_i \tag{10}$$

where K_{awa}, K_{aww}, K_{asoa} and K_{wsew} are mass transfer coefficients (MTCs) for air–water diffusion on the air side, air–water diffusion on the water side, air–soil diffusion on the air side and water–sediment diffusion on the water side, respectively (m/day); A_{aw}, A_{aso} and A_{wse} are the contact area between air and water, air and soil, and water and sediment, respectively (m^2); A_{ur} is the area of built-up regions (m^2); Z_{air}, Z_{water}, Z_{aero}, Z_{soil}, Z_{sus} and Z_{sedi} are the fugacity capacity of the air, water, aerosol, soil, suspended solid and sediment phases, respectively (mol/(m^3·Pa)); U_r, U_{dd}, U_{soff}, U_{woff}, U_{de} and U_{re} are the rain rate, dry deposition velocity, solid runoff rate from soil, water runoff from soil, sediment deposition rate and resuspension rate, respectively (m/day); Q is the scavenging ratio; v_{aero} is the volume fraction of the aerosol phase in the air compartment; L_{so} and L_{se} are the diffusion path length in soil and sediment, respectively (m); B_{ma}, B_{mw}, B_{mse} are molecular diffusivity in air, water and sediment, respectively (m^2/day); G_{wso} and G_i are the volume of water for irrigation and advection out of compartment i (m^3/day); and $\tau_{1/2i}$ is the chemical half-life in compartment i (day).

Z values in Equations (1)–(4) were estimated as follows:

$$Z_a = Z_{air} + v_{aero} \times Z_{aero} \tag{11}$$

$$Z_w = Z_{water} + v_{sus} \times Z_{sus} + v_{fi} \times Z_{fish} \tag{12}$$

$$Z_{so} = v_a \times Z_{air} + v_{wso} \times Z_{water} + v_{soil} \times Z_{soil} \tag{13}$$

$$Z_{se} = v_{wse} \times Z_{water} + v_{sedi} \times Z_{sedi} \tag{14}$$

where Z_{air}, Z_{aero}, Z_{water}, Z_{sus}, Z_{fish}, Z_{soil} and Z_{sedi} are the fugacity capacity of air, aerosol, water, suspended solid, fish, soil and sediment phases, respectively (mol/(m^3·Pa)); v_{aero} is the volume fraction of the aerosol phase in the air compartment; v_{sus} and v_{fi} are the volume fraction of the suspended solid phase and fish phase, respectively, in the water compartment; v_a, v_{wso} and v_{soil} are the volume fraction of the air, water and soil phase, respectively, in the soil compartment; and v_{wse} and v_{sedi} are the volume fraction of the water and sediment phase, respectively, in the sediment compartment.

Z values mentioned above were calculated as follows:

$$Z_{air} = \frac{1}{R \times T} \tag{15}$$

$$Z_{water} = \frac{S}{P \times M} \tag{16}$$

$$Z_{soil} = \frac{Y_{soil} \times K_{oc} \times Z_{water} \times \rho_{soil}}{1000} \tag{17}$$

$$Z_{sedi} = \frac{Y_{sedi} \times K_{oc} \times Z_{water} \times \rho_{sedi}}{1000} \tag{18}$$

$$Z_{sus} = \frac{Y_{sus} \times K_{oc} \times Z_{water} \times \rho_{sus}}{1000} \tag{19}$$

$$Z_{aero} = \frac{6000000 \times Z_{air}}{P} \tag{20}$$

$$Z_{fish} = v_{fish} \times K_{ow} \times Z_{water} \tag{21}$$

where R is the gas constant ((Pa·m^3)/(mol·K)); T is the absolute temperature of ambient air (K); S is the chemical aqueous solubility at 298 K (g/m^3); P is the chemical vapor pressure at 298 K (Pa); M is the chemical molar mass (g/mol); Y_{soil}, Y_{sedi} and Y_{sus} are the organic carbon mass fraction in soil, sediment and suspended solid phases, respectively; ρ_{soil}, ρ_{sedi} and ρ_{sus} are the density of soil, sediment and suspended solid phases, respectively (kg/m^3); K_{oc} is the organic carbon–water partition coefficient (L/kg); v_{fish} is mass fraction of lipids in fish; and K_{ow} is the octanol–water partition coefficient.

© 2015 by the authors; licensee MDPI, Basel, Switzerland. This article is an open access article distributed under the terms and conditions of the Creative Commons Attribution license (http://creativecommons.org/licenses/by/4.0/).
